# Mechanistic Insights into a Self-Management Intervention in Young Adults with Irritable Bowel Syndrome: A Pilot Multi-Omics Study

**DOI:** 10.3390/biomedicines13092102

**Published:** 2025-08-28

**Authors:** Weizi Wu, Jie Chen, Aolan Li, Ming-Hui Chen, Angela Starkweather, Xiaomei Cong

**Affiliations:** 1 Yale School of Nursing, Orange, CT 06477, USA; 2 College of Nursing, Florida State University, Tallahassee, FL 32306, USA; 3 Department of Statistics, University of Connecticut, Storrs, CT 06269, USA; 4 Division of Nursing Science, Rutgers School of Nursing, New Brunswick, NJ 08901, USA

**Keywords:** irritable bowel syndrome, multi-omics, self-management intervention, precision medicine

## Abstract

**Background:** Self-directed lifestyle modifications are essential for managing symptoms in individuals diagnosed with irritable bowel syndrome (IBS). This study incorporated longitudinal multi-omics profiling to estimate the mechanisms underlying responses to a nurse-led person-centered self-management intervention in young adults with IBS. **Methods:** This pre-post study was nested within a 12-week parent randomized controlled trial (NCT03332537). Biospecimens (stool and blood) and clinical outcomes were collected at baseline and post-intervention. Symptoms were assessed using the Brief Pain Inventory and PROMIS^®^ short forms. Host transcriptomic profiling was performed using RNA sequencing, and gut microbial composition was analyzed via 16S rRNA sequencing. Host transcriptomic co-expression and microbial co-abundance modules were identified via weighted gene co-expression network analysis. Associations between multi-omics modules and symptoms were evaluated using linear mixed-effect models. **Results:** Among the 20 participants, most were non-Hispanic (75%), White (75%), and female (65%). The intervention significantly reduced self-reported pain severity (*p* = 0.019) and pain interference (*p* = 0.013). Decreased associations were observed between pain phenotypes and a microbial module enriched in core metabolic pathways (interference: β = −4.7, *p* < 0.001; severity: β = −2.4, *p* = 0.02). Anxiety strengthened associations with host transcriptomic cellular energy metabolism pathways post-intervention (*p* < 0.05). The intervention attenuated associations between fatigue, sleep disturbance, and immune–inflammatory transcriptomic and microbial adaptation modules (*p* < 0.05). **Conclusions:** Findings suggest that the IBS self-management intervention induces symptom-specific biological responses, implicating distinct host–microbe pathways. Larger longitudinal studies are warranted to validate these omics-based symptom signatures.

## 1. Introduction

Irritable bowel syndrome (IBS), prevalent in young adults, is a disorder of brain-gut interaction characterized primarily by recurrent chronic abdominal pain, which is often accompanied and exacerbated by psychological comorbidities such as anxiety, depression, fatigue, and sleep disturbances [1,2]. IBS affects approximately 20% of the population in the United States, with annual direct healthcare costs and indirect productivity losses exceeding USD 21 billion [3,4,5]. Current clinical guidelines prioritize non-pharmacological interventions as first-line treatment for symptom management [6]. Comprehensive patient education facilitating self-directed lifestyle modifications, including dietary adjustment, stress management, and physical activity, represents a cornerstone strategy [7,8,9,10]. Despite these recommendations, intervention efficacy remains inconsistent and largely non-personalized, reflecting critical gaps in developing mechanism-based individualized IBS interventions.

Emerging evidence implicates dysregulation of the brain–gut-microbiome axis, including alterations of the central and autonomic nervous systems, as well as microbial dysbiosis, as key contributors of symptom persistence and severity in IBS [11,12]. Psychological stress exacerbates symptoms through activation of the hypothalamic–pituitary–adrenal axis, neurotransmitter balance disruption, and gut function alterations [13,14,15]. Visceral hypersensitivity, a hallmark feature of IBS [16,17], heightens sensitivity to normal and pathological stimuli, such as cramping and bloating. Experimental models suggest that such hypersensitivity may result from inflammatory processes in the colon, mediated by the upregulation of nociceptive receptors [18,19]. Consequently, the elevated somatic sensitivity demonstrated among IBS individuals has been associated with higher symptom burden [20]. However, the mechanistic insights of IBS symptoms remain fragmented, particularly regarding the interplay between host transcriptomics and the gut microbiome within clinical interventions, limiting the development of durable, personalized therapies.

Building on our prior work [21], this study uses a longitudinal multi-omics approach to investigate the biological underpinnings of clinical improvements from a 12-week IBS self-management program [22]. To uncover the complex, systems-level interactions that characterize IBS, we employed weighted gene co-expression network analysis (WGCNA). This network-based approach moves beyond individual genes or microbes to identify modules of co-regulated host transcripts and co-abundant microbial taxa [23]. We hypothesized that (a) distinct multi-omics modules would correlate with pain and its related psychoneurological comorbidities (anxiety, depression, fatigue, and sleep disturbance) at baseline and post-intervention, and (b) the intervention would modulate these multi-omics module–phenotype associations, thereby providing mechanistic insights into its therapeutic effects.

## 2. Materials and Methods

### 2.1. Study Design

This pre-post study utilized data from a 12-week parent randomized controlled trial (RCT, NCT03332537) of an IBS self-management intervention in young adults, conducted in the Northeastern U.S. from October 2016 to March 2019. The parent RCT enrolled 80 participants (intervention, *n* = 41; control, *n* = 39) who received online self-management modules, guided by the Individual and Family Self-Management Theory [24]; the intervention group also received nurse-led consultations and support. This analysis focuses on 20 participants from the intervention group who provided biological samples (blood and stool) at baseline and 12 weeks. This study has been approved by the University of Connecticut-Storrs Institutional Review Board (IRB # H16-152; approval date: 9 September 2016).

### 2.2. Participants’ Eligibility and Exclusion Criteria

Based on the parent protocol [21], participants were eligible if they met the following criteria: (1) aged 18–29 years; (2) had a clinician-confirmed IBS diagnosis (Rome III or IV criteria); (3) reported active IBS-related pain, defined as ≥3 episodes per week over the past month; (4) had internet access and were able to read and speak English; and (5) were willing to participate in the study.

Individuals were excluded if they had: (1) comorbid chronic pain conditions (e.g., fibromyalgia, chronic pelvic pain); (2) other gastrointestinal disorders (e.g., inflammatory bowel disease, celiac disease); (3) systemic illnesses (e.g., diabetes, hepatitis, HIV); (4) severe psychiatric conditions (e.g., bipolar disorder); (5) current pregnancy or were within three months postpartum; (6) regular use of antibiotics, probiotics, opioids, or iron supplements (>3 times per week) within the past month; or (7) skin injuries or lesions on the non-dominant hand that could interfere with biospecimen collection.

### 2.3. Measures

Demographic and clinical characteristics, including sex, age, race, ethnicity, education, and employment status, were collected through structured questionnaires. Pain severity and interference were assessed using the Brief Pain Inventory (BPI) short form, which quantifies the average of pain intensity and functional interference over the past week on 0–10 Likert scales [25]. Anxiety, depression, fatigue, and sleep disturbance were measured using validated Patient-Reported Outcomes Measurement Information System (PROMIS^®^) short-form instruments (v1.0) [26].

### 2.4. Stool Sample Collection and Gut Microbiome Sequencing

Participants were instructed to collect stool samples at home using the OMNIgene-GUT (OMR-200) collection kit (DNA Genotek Inc., Ottawa, ON, Canada) and mail back the samples within 1 week after the lab visits. Samples were stored at −80 °C until processing. DNA was extracted from 0.25 g of each stool sample using the MagAttract PowerSoil Pro DNA kit (Qiagen, Inc. Germantown, MD, USA). The V4 region of the 16S rRNA gene was then amplified using 515F and 806R primers. The final library was cleaned and sequenced on an Illumina MiSeq platform (2 × 250 bp paired-end reads). Full details regarding primer sequences, PCR conditions, and the library preparation workflow are available in the Supplementary Materials. 

### 2.5. Blood Sample Collection and RNA Sequencing

Peripheral blood samples were collected at baseline and 12-week follow-up visits, using PAXgene^®^ Blood RNA tubes. All procedures were performed by trained registered nurses following strict sterile techniques. The samples were stored at −80°C until RNA extraction at the institute’s Biobehavioral Lab. RNA sequencing assay was performed at the institute’s Genome Innovation Center. RNA extraction isolated total RNA from blood samples using phenol-chloroform for high purity and integrity. DNase treatment followed to remove genomic DNA contamination. The main RNA seq includes library preparation and sequencing. Following the manufacturer’s protocol (Illumina, San Diego, CA, USA), libraries were prepared using the Illumina TruSeq Stranded mRNA Kit and sequenced on an Illumina NextSeq 500/550 platform. Target read depth was achieved per sample with paired-end 75bp reads. Then, the paired-end reads were obtained and stored in Xanadu Cluster, a secure platform hosted by the study institute’s Computational Biology Core.

### 2.6. Data Analysis

The data analysis was performed using R version 4.2.1 statistical packages. The demographic characteristics, self-reported pain, and other symptoms were summarized with frequency and percentage for categorical variables and the mean and SD for continuous variables. Paired sample *t*-tests assessed differences in self-reported symptoms at baseline and after the intervention.

Both omics workflows integrated paired sample longitudinal changes and intervention responses to link microbial and transcriptional networks with clinical phenotypes. For microbiome analysis, raw 16S rRNA sequencing reads were processed using Mothur 1.43.0 [27,28] (MiSeq pipeline) to generate taxonomic profiles and alpha/beta diversity metrics, as detailed previously [29]. To control for sequencing depth, all samples were rarefied to 10,000 reads per sample. Alpha diversity (inverse Simpson, Shannon, and observed richness) and beta diversity (Bray–Curtis) were calculated on the rarefied dataset. Group differences in alpha diversity were assessed using Wilcoxon signed-rank tests, while beta diversity was compared using PERMANOVA with 1000 permutations. Longitudinal weighted gene co-expression network analysis (WGCNA) identified co-abundance microbial modules. Microbial functional profiling was performed using Tax4Fun2, which predicted metabolic pathway abundances based on Kyoto Encyclopedia of Genes and Genomes (KEGG) orthologs inferred from 16S rRNA gene sequences [19]. For WGCNA, filtering was applied to remove taxa with a detection threshold of 0.1% and a prevalence threshold of 5%. The resulting dataset was centered log-ratio transformed and used for module detection. Functional enrichment analysis was then conducted for each WGCNA module to identify KEGG functional pathways that were overrepresented within specific microbial modules. For transcriptomics, RNA-seq data underwent quality control (fastqc, multiqc), trimming (fastp), alignment to hg38 (HISAT2), gene quantification (HTseq-count), and batch correction (ComBat-seq). Normalized counts (DESeq2) were analyzed via WGCNA to define co-expressed transcriptional modules. Functional enrichment analyses were performed on transcriptomics modules using Gene Ontology (GOseq) and KEGG pathway (KEGG) analyses, with outcomes depicted in enrichment dot plots. Linear mixed-effect models evaluated associations between omics-derived modules and self-reported symptoms, as well as the intervention-induced modulations, with significance thresholds set at two-sided *p* < 0.05. Pearson correlation analyses were used to explore interrelationships between omics modules further. Module–trait associations from WGCNA were reported as unadjusted *p*-values. KEGG enrichment analyses were corrected for multiple testing using the Benjamini–Hochberg false discovery rate, and adjusted q-values < 0.05 were considered significant. Results were visualized using heatmaps generated with the ggplot2 package [30].

## 3. Results

### 3.1. Demographics, Clinical Symptoms, and Microbial Diversity Profiles

Among the twenty participants included in the current analysis, most participants were non-Hispanic (75%), White (75%), female (65%), and single (90%). Detailed demographic and clinical characteristics are presented in Table 1. Post-intervention assessments revealed a decrease in all self-reported symptom scores compared to baseline, with statistically significant reductions in pain severity (*p* = 0.019) and pain interference (*p* = 0.013) (Table 2).

Microbiome analyses revealed no significant differences in alpha diversity indices between visits (Table 3). Similarly, beta diversity analysis indicated no significant differences in community composition between visits (PERMANOVA).

### 3.2. Multi-Omics Module Function Characteristics at Baseline and Post-Intervention

Weighted co-expression network analysis (WGCNA) identified nine host co-expression transcriptomic and eight microbial co-abundance modules (FDR < 0.05) from multi-omics profiles (full annotations in Tables S1 and S2, Figures S1 and S2).

Host transcriptomic modules were stratified into three primary mechanistic axes: (1) Immune–inflammatory regulation axes comprised multiple modules associated with defense mechanisms. The magenta module was enriched in key inflammatory pathways, including NOD-like receptor signaling (hsa04621), NF-κB signaling (hsa04064), and TNF signaling (hsa04668). The green module reflected innate immune activation, enriched in neutrophil extracellular trap formation (hsa04613) and phagosome pathways (hsa04145), while the blue module was enriched in B-cell receptor signaling (hsa04662) and chemokine signaling (hsa04062). Additionally, the red and pink modules demonstrated roles in antiviral defenses, with enrichment in antigen presentation (hsa04612), lysosomal function (hsa04142), and efferocytosis (hsa04148). (2) The metabolic regulation axis included the turquoise and yellow modules, which were enriched in pathways related to cellular energy metabolism, particularly oxidative phosphorylation (hsa00190) and reactive oxygen species (ROS) metabolism (hsa05208). (3) Other homeostatic regulation axes captured broader physiological regulation, including the black and brown modules enriched in hormonal signaling pathways, including thyroid hormone signaling (hsa04919), growth hormone synthesis, secretion, and action (hsa04935), and oxytocin signaling (hsa04921).

The microbial co-abundance modules were classified into four primary functional categories based on pathway enrichment profiles as described hereinafter: (1) Core metabolism and specific nutrient utilization: this category encompasses fundamental metabolic processes and specialized substrate catabolism. The pink module showed specificity for the processing of complex carbohydrates and sphingolipids. The brown module was enriched in amino acid biosynthesis (map01230), phosphate transport systems (map02060), and TCA cycle activity (map00020). (2) Quorum sensing and community coordination: represented by the red and black modules, this category highlights microbial communication and collective behavior. The red module was enriched for quorum sensing (map02024) and Caulobacter cell cycle regulation (map04112). (3) Environmental adaptation and microbial defense: primarily characterized by the turquoise module, this category reflects microbial strategies for environmental sensing, colonization, and survival under stress conditions. Enriched pathways included bacterial chemotaxis (map02030), biofilm formation (map02025/map02026), and antimicrobial resistance (map01503/map01501). (4) Carbohydrate metabolism and nutrient–signal integration: comprising the blue, green, and yellow modules, this category centers on the metabolism of diverse carbohydrates and the integration of nutrient processing with signaling pathways. The blue module was enriched for galactose metabolism (map00052), amino sugar degradation (map00520), fatty acid biosynthesis (map00061), and glycan degradation (map00511), which were co-enriched with quorum sensing (map02024).

### 3.3. Associations Between Symptom Phenotypes and Multi-Omics Modules at Baseline

**Figure 1:**
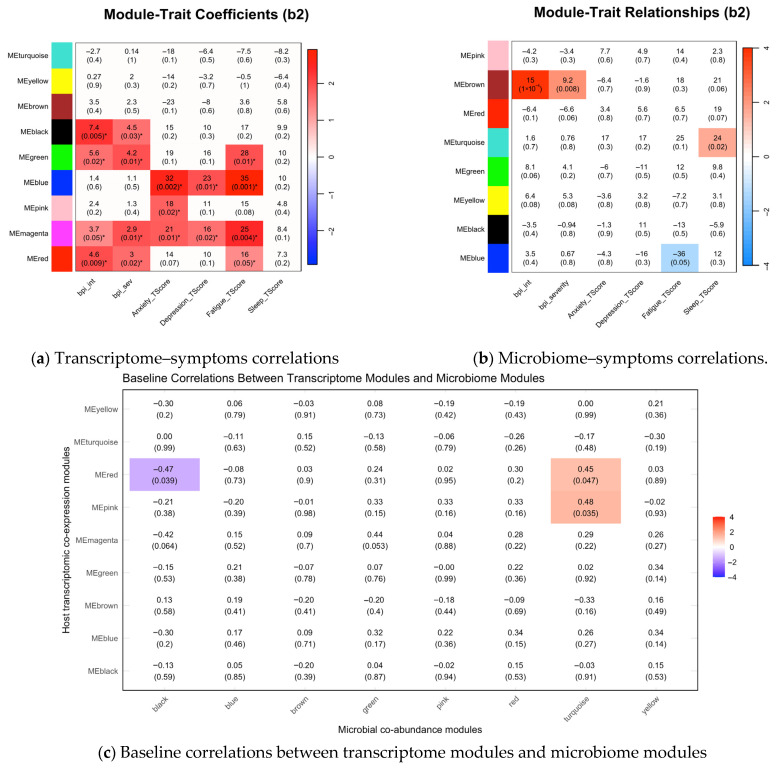
Heatmaps of baseline correlations between symptoms, transcriptomic co-expression modules, and microbiome co-abundance modules. (**a**,**b**) Linear mixed-effects model coefficients for module eigengene (ME) effects on symptoms (baseline, adjusted for patient-level random effects). (**c**) Pearson correlation coefficients between modules. White indicates non-significant associations (*p* ≥ 0.05). Color intensity reflects statistical significance; red = positive, blue = negative. These baseline data reveal distinct multi-omic signatures for different symptoms; for example, pain is associated with both host and microbial modules (e.g., host black and microbial brown modules), whereas anxiety is primarily linked to host transcriptomic modules (e.g., blue and magenta modules), and sleep disturbance is only associated with a single microbial module (turquoise module). Furthermore, the cross-omic analysis (**c**) reveals that the turquoise microbial module has a significant positive correlation with the host red transcriptomic module. * *p* < 0.05.

At baseline (Figure 1), symptoms showed distinct associations with host transcriptomic modules related to endocrine, immune, and inflammatory pathways (Figure 1a). Pain interference (β = 7.4, *p* = 0.01) and severity (β = 4.5, *p* = 0.03) were significantly associated with the thyroid hormone signaling pathway (black module). Across symptom domains, all phenotypes except sleep disturbance were linked to immune–inflammatory processes, including innate immune responses (green), leukocyte-mediated immunity (blue), antigen processing and presentation (pink), key inflammatory pathways (magenta), and antiviral defense mechanisms (red) (all *p* < 0.05). For instance, anxiety was positively associated with leukocyte-mediated immunity (blue: β = 32, *p* = 0.002), antigen processing/presentation (pink: β = 18, *p* = 0.02), and inflammatory pathways (magenta: β = 21, *p* = 0.01). A comprehensive summary of these associations is provided in Figure 1a.

For microbial modules (Figure 1b), pain interference and severity were strongly positively associated with central metabolism (brown module: β = 15, *p* < 0.001, and β = 9.2, *p* = 0.01, respectively). Fatigue was negatively associated with nutrient metabolism (blue: β = −36, *p* = 0.05), while sleep disturbance was positively linked with environmental adaptation (turquoise: β = 24, *p* = 0.02). Full microbial–symptom associations are shown in Figure 1b.

Baseline cross-omics correlations (Figure 1c) showed positive associations between microbial environmental adaptation (turquoise microbial module) and host antigen presentation (pink transcriptomic module; r = 0.48, *p* = 0.04) and antiviral defense (red transcriptomic module; r = 0.45, *p* = 0.047). Conversely, host antiviral defense (red transcriptomic module) negatively correlated with microbial quorum sensing/community coordination (black microbial module; r = −0.47, *p* = 0.04).

### 3.4. Associations Between Symptom Phenotypes and Multi-Omics Modules Post-Intervention

**Figure 2:**
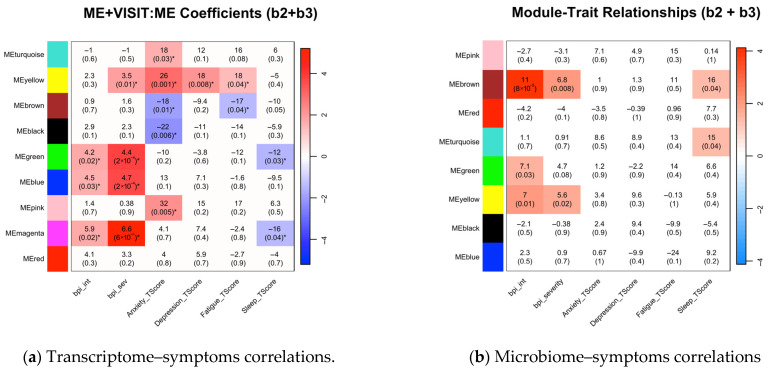

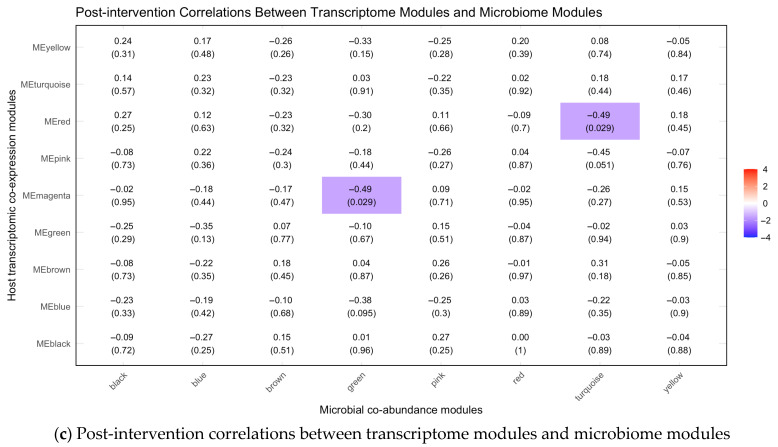
Heatmaps of post-intervention correlations between symptoms, transcriptomic co-expression modules, and microbiome co-abundance modules. (**a**,**b**) Displayed coefficients from linear mixed-effects models represent the module eigengene (ME) effect on symptoms at follow-up, calculated as b2 + b3 (accounting for the main ME effect and its interaction with time). (**c**) Pearson correlation coefficients between modules. White indicates non-significant associations (*p* ≥ 0.05). Color intensity reflects statistical significance; red = positive, blue = negative. These post-intervention results show a shifting landscape of symptom–module associations, where some baseline correlations persist while new ones emerged. For instance, the link between pain and several modules remains significant (e.g., host magenta and microbial brown modules), while a new, strong association developed between anxiety and the host turquoise and yellow modules. The cross-omic analysis also reveals new negative correlations post-intervention, such as between the host magenta and microbial green modules (**c**). * *p* < 0.05.

Post-intervention (Figure 2), pain interference and severity remained positively associated with immune–inflammatory pathways (green, magenta, and blue transcriptomic modules; *p* < 0.05) (Figure 2a). Anxiety positively associated with cellular energy metabolism (turquoise: β = 18, *p* = 0.03; yellow: β = 26, *p* = 0.001) and negatively with hormonal signaling (brown: β = −18, *p* = 0.01; black: β = −22, *p* = 0.01). Fatigue showed similar positive associations with energy metabolism (yellow: β = 18, *p* = 0.04) and negative associations with hormonal signaling (brown: β = −17, *p* = 0.04). Sleep disturbance was negatively associated with immune–inflammatory pathways (green: β = −12, *p* = 0.03; magenta: β = −16, *p* = 0.04). Detailed post-intervention associations are visualized in Figure 2a.

Microbial analyses (Figure 2b) showed continued strong positive associations for pain interference and severity with central metabolism (brown module: β = 11, *p* = 8 × 10^−5^, and β = 6.8, *p* = 0.01, respectively). Pain interference was further associated with carbohydrate metabolism/nutrient-sensing (green: β = 7.1, *p* = 0.03; yellow: β = 7.0, *p* = 0.01), while pain severity was associated with the yellow module (β = 5.6, *p* = 0.02). Sleep disturbance positively correlated with central metabolism (brown: β = 16, *p* = 0.04) and environmental adaptation (turquoise: β = 24, *p* = 0.02).

Post-intervention cross-omics (Figure 2c) identified negative correlations between host antiviral defenses (red transcriptomic module) and microbial environmental adaptation (turquoise microbial module; r = −0.49, *p* = 0.03), and between host inflammatory signaling (magenta transcriptomic module) and microbial carbohydrate metabolism (green microbial module; r = −0.49, *p* = 0.03).

### 3.5. Intervention-Induced Modulation of the Associations Between Symptoms and Multi-Omics

**Figure 3:**
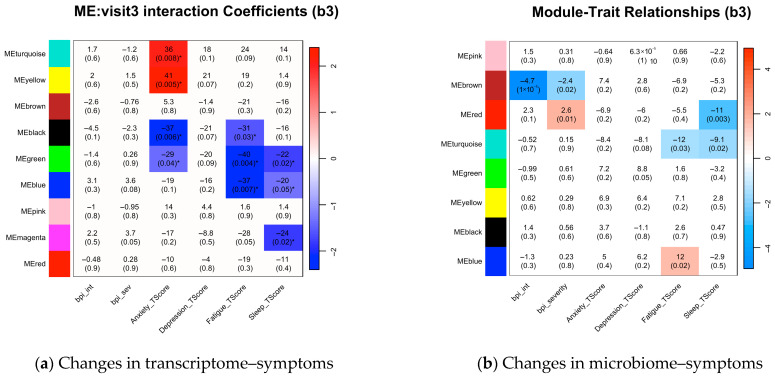
Heatmaps of intervention-induced changes in symptom correlations with transcriptomic and microbiome co-expression modules. (**a**,**b**) Values shown are β coefficients representing the module eigengene (ME) × visit interaction effect (b3), indicating how the ME–symptom association changes over time; a positive β signifies a strengthened association. White indicates non-significant interaction effects (*p* ≥ 0.05). Color intensity reflects statistical significance of the interaction; red = positive β, blue = negative β. These results highlight the intervention’s symptom-specific effects, such as significantly attenuating the link between pain and the microbial brown module, while strengthening the association between anxiety and the host turquoise and yellow modules. * *p* < 0.05.

Linear mixed-effects models (time-by-module interactions) demonstrated significant intervention-related modulation of symptom–module associations across multi-omics layers (Figure 3).

At the transcriptomic level (Figure 3a), anxiety showed strengthened associations with host cellular energy metabolism (turquoise: β = +36, *p* = 0.01; yellow: β = +41, *p* = 0.01) and attenuated associations with thyroid hormone signaling (black: β = −37, *p* = 0.01) and innate immune activation (green: β = −49, *p* = 0.004). Similarly, fatigue showed reduced associations with hormonal signaling (black: β = −31, *p* = 0.03), innate immunity (green: β = −49, *p* = 0.004), and leukocyte-related pathways (blue: β = −37, *p* = 0.01). Sleep disturbance also demonstrated diminished associations with immune–inflammatory modules (green: β = −22, *p* = 0.02; blue: β = −20, *p* = 0.047; magenta: β = −24, *p* = 0.02).

At the microbial level (Figure 3b), pain interference and severity showed attenuated associations with central metabolism (brown: β = −4.7, *p* < 0.01 and β = −2.4, *p* = 0.02, respectively). Pain severity also showed a strengthened association with microbial communication pathways (red: β = +2.6, *p* = 0.01). Fatigue had reduced linkage to microbial environmental adaptation (turquoise: β = −12, *p* = 0.03) and increased association with carbohydrate metabolism (blue: β = +12, *p* = 0.02). Sleep disturbance exhibited decreased associations with microbial communication and adaptation pathways (red: β = −11, *p* = 0.003; turquoise: β = −9.1, *p* = 0.02).

## 4. Discussion

This study provides mechanistic insights indicating that the self-management interventions elicited symptom-specific biological responses, supporting distinct molecular pathways underlying clinical improvements. Findings suggested that the intervention significantly reduced self-reported pain severity and interference. At both baseline and post-intervention, pain was associated with distinct transcriptomic modules enriched in immune–inflammatory pathways. Although the intervention appeared to modulate these associations, the changes did not reach statistical significance, potentially due to the limited 12-week follow-up period and small sample size. Notably, the association between pain and microbiota modules involved in core metabolic functions was significantly attenuated following the intervention, suggesting microbial contributions to pain modulation, which aligns with previous findings [2,11]. Anxiety and depression were linked predominantly to transcriptomic modules before and after the intervention. Anxiety exhibited strengthened associations with modules enriched in cellular energy metabolism, indicating transcriptomic contributions to anxiety. Fatigue/sleep reflected an integrated recalibration of host immune–inflammatory transcriptome and gut microbiome environmental adaptation interactions. Despite sample size and follow-up duration limitations, this study leveraged a multi-omics framework to uncover testable hypotheses, laying foundational evidence for advancing precision and personalized symptom management strategies in IBS [17].

Pain, the hallmark clinical symptom of IBS, demonstrated significant improvement following the intervention [21]. The associations between pain, host-derived immune–inflammatory transcriptomic signatures, and metabolic dysregulations were observed at baseline and post-intervention. These were consistent with prior mechanistic findings in IBS, marked by increased cellular immune activation, elevated proinflammatory cytokines, mucosal barrier disruption, intestinal permeability, and heightened visceral hypersensitivity compared to the healthy control population [29,31]. By the 12-week follow-up, the associations between pain severity, pain interference, and core microbiota metabolic pathways were notably diminished. This shift may be partially attributed to the dietary modifications introduced during the intervention, particularly the increased intake of soluble fiber and adherence to a low-fermentable oligo-, di-, monosaccharides, and polyols (low-FODMAP) diet [32,33]. This evidence-based nutritional approach has been shown to attenuate microbial fermentation of carbohydrates, reduce abdominal bloating, support intestinal fluid homeostasis, and thereby alleviate pain in individuals with IBS [34,35]. Our findings also revealed a significantly positive association between pain and modules enriched in carbohydrate metabolism following the intervention. Intervention strengthened the association between pain severity and microbial ecosystem coordination pathways, such as microbiota enriched in quorum sensing (map02024). One biologically plausible mechanism has been proposed: quorum sensing, a form of bacterial communication, mediates visceral hypersensitivity enhancement through intensified communication between gut microbiota and sensory neurons [36]. While it remains to be fully elucidated, these microbial coordination modules may represent early biomarkers of IBS pain chronicity and have potential as therapeutic targets. Future studies are needed to validate this finding, including longitudinal multi-omics and preclinical models.

Anxiety was associated with distinct immune–inflammatory transcriptomic signatures at baseline, including enrichment in pathways related to leukocyte-mediated immunity and inflammatory responses, consistent with the known role of neuroimmune crosstalk in stress-related psychopathology [37,38]. Although not statistically significant, the observed changes suggested a trend toward immunological recalibration, which warrants further study. Following the intervention, the association shifted, with anxiety showing a stronger association with transcriptomic modules for oxidative phosphorylation and reactive oxygen species (ROS) metabolism. This is highly relevant to pain pathophysiology, as these pathways are implicated not only in anxiety regulation but also in modulating neuronal excitability and central sensitization, which are key mechanisms in maintaining chronic pain states [39]. Notably, oxidative phosphorylation, a cellular process that generates ATP through the breakdown of glucose and other substrates, typically reflects heightened energy demand [40]. Furthermore, the role of ROS is dose- and site-dependent: at low levels, ROS facilitate adaptive cellular responses to stress through signaling, whereas high levels can trigger oxidative stress [41]. The intervention’s relaxation-focused components may explain the strengthened association between these transcriptomic pathways and anxiety. These practices, including belly breathing and mindfulness, are designed to stimulate the vagal nerve. This stimulation helps shift the body from a sympathetic ‘fight-or-flight’ state to a parasympathetic state of rest and restoration [34,42]. Mechanistically, these practices are thought to exert top-down modulation of limbic system hyperactivity, reduce glucocorticoid receptor resistance, and restore rhythmic functioning of the HPA axis [43], ultimately lowering oxidative stress burden. However, as this study did not quantify absolute levels of oxidative phosphorylation and ROS or localize their activity, the observed regulation may represent either a reduction in metabolic stress due to behavioral deactivation of the stress response or a maladaptive process in response to chronic stress. Nonetheless, ROS-related biomarkers have increasingly been utilized in prognosis and therapeutic guidance in stress-related diseases and disorders [44]. Additionally, oxidative stress markers have demonstrated emerging utility in diagnosing inflammatory bowel disease and monitoring treatment responses [45]. Therefore, further studies are warranted to characterize oxidative stress within the context of IBS.

Fatigue and sleep disturbances, two common and burdensome comorbidities in chronic pain, demonstrated an integrated response involving both microbial and host transcriptomic expression, reflecting their complex pathophysiology in IBS. At baseline, sleep disturbances were positively associated with microbial environmental adaptation strategies, primarily chemotaxis, biofilm formation, and antimicrobial resistance, while nutrient metabolism pathways were negatively correlated with fatigue. These features are indicative of gut ecosystem instability and dysbiosis [46]. Following the intervention, these maladaptive microbial–host interactions were markedly attenuated, and inflammatory signatures were reduced across fatigue- and sleep-related molecular networks [47]. This suggests that promoting health-supportive behaviors, such as improving sleep hygiene and physical activity, through the intervention contributed to microbial community stabilization. Via the gut–microbiome–brain axis, such microbial recalibration may restore gut barrier integrity and rebalance immune–inflammatory pathways, which in turn can improve pain outcomes by reducing inflammatory load and central nervous system hyperexcitability [48,49,50].

Several limitations warrant consideration. First, the relatively small sample size (*n* = 20), limited population diversity, and lack of stratification by IBS subtypes constrain the generalizability of our findings. Given the limited statistical power, our findings should be regarded as hypothesis-generating and require validation in larger, more definitive longitudinal studies. In addition, incorporating metataxonomic profiling and differential gene expression analyses could further strengthen the results. Additionally, the 12-week follow-up period may be insufficient to fully capture systemic immune recalibration, which may require a longer timeframe to emerge. Future studies should extend the follow-up window to 6 months or 1 year.

## Figures and Tables

**Table 1:** Demographic characteristics of young adults with irritable bowel syndrome (*n* = 20).

	Mean	SD
Age	22.05	2.74
Year of IBS diagnosis	2.25	1.89
	**N**	**Percentage**
Sex		
Female	13	65.0
Male	7	35.0
Race		
White	15	75.0
Asian	2	10.0
Black or African American	3	15.0
Ethnicity		
Not Hispanic or Latino	15	75.0
Hispanic or Latino	3	15.0
Not reported	2	10.0
Education		
High school or below	0	0
College or associate degree	11	55.0
Bachelor’s degree	5	25.0
Graduate or higher	4	20.0
Employment Status		
Student	13	65.0
Working now	7	35.0
Unemployed or other	0	0
Marital Status		
Never married	18	90.0
Married	2	10.0

**Table 2:** Difference in self-reported symptoms at baseline and post-intervention.

Self-Reported Symptom	Baseline (M ± SD)	Post-Intervention (M ± SD)	95% CI	*p*
Pain interference	2.65 ± 2.77	1.52 ± 1.88	[−1.99, −0.27]	0.013 *
Pain severity	3.05 ± 2.14	2.29 ± 1.78	[−1.39, −0.14]	0.019 *
Anxiety	58.76 ± 8.30	56.43 ± 9.02	[−6.28, 1.61]	0.231
Depression	52.53 ± 8.07	50.52 ± 8.48	[−5.03, 1.02]	0.181
Fatigue	53.53 ± 9.63	52.91 ± 10.98	[−4.44, 3.19]	0.735
Sleep disturbance	49.90 ± 5.84	48.40 ± 5.15	[−4.1, 1.1]	0.243

Note. A higher score presented higher symptom levels for all symptom items. M. mean; SD. standard deviation. CI. confidence interval. * *p* < 0.05 by paired samples *t*-tests.

**Table 3:** Difference in microbiome diversity at baseline and post-intervention.

Metric	Baseline (M ± SD)	Post-Intervention (M ± SD)	Wilcox *p*
Alpha Diversity			
invsimpson	9.51 ± 4.64	9.72 ± 5.2	0.756
shannon	2.95 ± 0.68	3.01 ± 0.63	0.596
sobs	228.69 ± 57.63	238.31 ± 52.2	0.202
Beta Diversity			
Within-group Bray–Curtis distance	0.67 ± 0.18	0.65 ± 0.16	0.482

Note. M. mean; SD. standard deviation. * *p* < 0.05 by Wilcoxon signed-rank tests.

## Data Availability

The raw sequence data were archived in NCBI, https://submit.ncbi.nlm.nih.gov/subs/sra/SUB8914789/. Requests to access these datasets should be directed to xiaomei.cong@yale.edu. To safeguard participant privacy, the following protocols regulate access to the datasets: de-identified clinical data, transcriptomic and microbiome analysis code, and computational workflows can be made available upon reasonable request to the corresponding author (xiaomei.cong@yale.edu), contingent upon the approval of a Data Transfer Agreement by the Yale University Ethics Committee. The study protocol has been published and can access at https://pmc.ncbi.nlm.nih.gov/articles/PMC6415297/. Individual participant data will not be shared.

## Supplementary Materials

The following supporting information can be downloaded at: https://www.mdpi.com/article/10.3390/biomedicines13092102/s1, Table S1. Summary of function pathway analysis for transcriptomic co-expression modules Table S2. Summary of function pathway analysis for microbiome co-abundance modules. Figure S1. Kyoto Encyclopedia of Genes and Genomes (KEGG) pathway enrichment analysis of transcriptomic modules. Figure S2. Kyoto Encyclopedia of Genes and Genomes (KEGG) Pathway enrichment analysis of microbiome modules.

## Abbreviations

The following abbreviations are used in this manuscript:

IBS	Irritable bowel syndrome
WGCNA	Weighted gene co-expression network analysis
RCT	Randomized controlled trial
PROMIS	Patient-Reported Outcomes Measurement Information System
KEGG	Kyoto Encyclopedia of Genes and Genomes
ROS	Reactive oxygen species
FODMAP	Low-fermentable oligo-, di-, monosaccharides, and polyols
