# Supplementary material for: Mechanistic Insights into a Self-Management Intervention in Young Adults with Irritable Bowel Syndrome: A Pilot Multi-Omics Study
Source: Biomedicines. 2025 Aug 28;13(9):2102. doi: 10.3390/biomedicines13092102 (PMC12467349; doi:10.3390/biomedicines13092102)
Supplement: Supplementary file 1 [file biomedicines-13-02102-s001.zip › biomedicines-3769897-supplementary.pdf]

Supplementary Information

Table S1. Summary of function pathway analysis for transcriptomic co-expression modules.

Modules (Gene counts)	Function summary	GO enrichment analysis*	KEGG analysis*
**Immune-inflammatory**			
Magenta Module (N=138)	Immune & Inflammatory Responses and external stimuli.	GO:0051607: defense response to virus<br>GO:0002366: leukocyte activation involved in immune response<br>GO:0002263: cell activation involved in immune response<br>GO:0050729: positive regulation of inflammatory response<br>GO:0002221: pattern recognition receptor signaling pathway	hsa05132: Salmonella infection<br>hsa05164: Influenza A<br>hsa04621: NOD-like receptor signaling pathway<br>hsa04064: NF-kappa B signaling pathway<br>hsa04668: TNF signaling pathway
Green Module (N=280)	Innate immune responses, signaling pathways, and enzymatic regulation	GO:0051345: positive regulation of hydrolase activity<br>GO:0034134: toll-like receptor 2 signaling pathway<br>GO:0002218: activation of innate immune response<br>GO:0043087: regulation of GTPase activity<br>GO:0032868: response to insulin	hsa04613: Neutrophil extracellular trap formation<br>hsa05152: Tuberculosis<br>hsa04380: Osteoclast differentiation<br>hsa04145: Phagosome<br>hsa05161: Hepatitis B
Blue Module (N=474)	Leukocyte-Mediated Immunity	GO:0002703: regulation of leukocyte mediated immunity<br>GO:0002366: leukocyte activation involved in immune response<br>GO:0043299: leukocyte degranulation<br>GO:0002263: cell activation involved in immune response<br>GO:0043300: regulation of leukocyte degranulation	hsa04380: Osteoclast differentiation<br>hsa04670: Leukocyte transendothelial migration<br>hsa04662: B cell receptor signaling pathway<br>hsa04062: Chemokine signaling pathway<br>hsa04145: Phagosome
Red Module (N=173)	Antiviral Defense and Regulation of Viral Processes	GO:0051607: defense response to virus<br>GO:0050792: regulation of viral process<br>GO:0045071: negative regulation of viral genome replication<br>GO:1903900: regulation of viral life cycle<br>GO:0048525: negative regulation of viral process	hsa05164: Influenza A<br>hsa04621: NOD-like receptor signaling pathway<br>hsa05162: Measles<br>hsa05171: Coronavirus disease - COVID-19<br>hsa05168: Herpes simplex virus 1 infection
Pink Module (N=153)	Antigen processing and presentation	GO:0070371: ERK1 and ERK2 cascade<br>GO:0046598: positive regulation of viral entry into host cell<br>GO:0075294: positive regulation by symbiont of entry into host<br>GO:1901136: carbohydrate derivative catabolic process<br>GO:0070372: regulation of ERK1 and ERK2 cascade	hsa04612: Antigen processing and presentation<br>hsa04142: Lysosome<br>hsa03250: Viral life cycle - HIV-1<br>hsa04979: Cholesterol metabolism<br>hsa04148: Efferocytosis
**Metabolic pathway**			
Turquoise Module (N=492)	Housekeeping processes critical for basal cellular	GO:0002181: cytoplasmic translation<br>GO:0006119: oxidative phosphorylation<br>GO:0042254: ribosome biogenesis	hsa03010: Ribosome<br>hsa05171: Coronavirus disease - COVID-19<br>hsa00190: Oxidative phosphorylation

Yellow Module (N=292)	metabolism and ener gy homeostasis.	GO:0009060: aerobic respiration<br>GO:0042773: ATP synthesis coupled electron transport	hsa05208: Chemical carcinogenesis - reactive oxygen species<br>hsa05012: Parkinson disease
	Energy metabolism	GO:0015669: gas transport<br>GO:0042744: hydrogen peroxide catabolic process<br>GO:0015671: oxygen transport<br>GO:0019755: one-carbon compound transport<br>GO:0042743: hydrogen peroxide metabolic process	hsa05012: Parkinson disease<br>hsa00190: Oxidative phosphorylation<br>hsa05208: Chemical carcinogenesis - reactive oxygen species<br>hsa05144: Malaria<br>hsa05022: Pathways of neurodegeneration - multiple diseases
**Other homeostatic regulators**			
Black Module (N=160)	Dynamic Cellular Processes, Homeostasis, and Thyroid hormone signaling pathway	GO:0032984: protein-containing complex disassembly<br>GO:0048511: rhythmic process<br>GO:0045765: regulation of angiogenesis<br>GO:1901342: regulation of vasculature development<br>GO:0007623: circadian rhythm	hsa04919: Thyroid hormone signaling pathway<br>hsa05167: Kaposi sarcoma-associated herpesvirus infection<br>hsa01521: EGFR tyrosine kinase inhibitor resistance<br>hsa04935: Growth hormone synthesis, secretion and action<br>hsa05163: Human cytomegalovirus infection
Brown Module (N=358)	Regulatory processes involving s ignal transduction, hormon e responses, and metabolic adaptation.	GO:0018105: peptidyl-serine phosphorylation<br>GO:0018209: peptidyl-serine modification<br>GO:0071375: cellular response to peptide hormone stimulus<br>GO:0032869: cellular response to insulin stimulus<br>GO:0046777: protein autophosphorylation	hsa05205: Proteoglycans in cancer<br>hsa04921: Oxytocin signaling pathway<br>hsa00770: Pantothenate and CoA biosynthesis<br>hsa04068: FoxO signaling pathway<br>hsa05219: Bladder cancer

* Top 5 based on the adjusted P value for each term.

Table S2. Summary of function pathway analysis for microbiome co-abundance modules.

Modules (OTU counts)	Function summary	ID*	Description	FDR
**Core Metabolism and Specific Nutrient Utilization**				
Pink (N=21)	Complex Carbohydrate and Sphingolipid Processing	map00511	Other glycan degradation	1.59×10^{-6}
		map00052	Galactose metabolism	0.000
		map00520	Amino sugar and nucleotide sugar metabolism	0.000
		map00600	Sphingolipid metabolism	0.001
		map00531	Glycosaminoglycan degradation	0.003
Brown (N=44)	Central Metabolic Network Coordination and Community Regulation	map01230	Biosynthesis of amino acids	2.29×10^{-6}
		map02060	Phosphotransferase system (PTS)	4.65×10^{-5}
		map00071	Fatty acid degradation	0.002
		map01210	2-Oxocarboxylic acid metabolism	0.004
		map02024	Quorum sensing	0.022
**Quorum Sensing and Community Coordination**				
Red (N=32)	Sociometabolic Resilience and Coordinated Adaptation	map02024	Quorum sensing	0.000
		map04112	Cell cycle - Caulobacter	0.004
		map03430	Mismatch repair	0.011
		map02030	Bacterial chemotaxis	0.023
		map00052	Galactose metabolism	0.050
Black (N=31)	Microbial Niche Adaptation and Metabolic Coordination	map04112	Cell cycle - Caulobacter	0.000
		map00511	Other glycan degradation	0.001
		map00052	Galactose metabolism	0.001
		map02024	Quorum sensing	0.007
		map00270	Cysteine and methionine metabolism	0.008
**Environmental Adaptation and Microbial Defense**				
Turquoise (N=115)	Coordinated Environmental Navigation and Metabolic Adaptation	map02030	Bacterial chemotaxis	5.82×10^{-11}
		map02024	Quorum sensing	2.20×10^{-9}
		map04112	Cell cycle - Caulobacter	0.004
		map02026	Biofilm formation - Escherichia coli	0.004
		map00052	Galactose metabolism	0.011
**Carbohydrate Metabolism and Nutrient-Signal Integration**				
Blue (N=61)	Nutrient Metabolism and Community Signaling for Ecological Adaptation	map00052	Galactose metabolism	1.16×10^{-5}
		map00520	Amino sugar and nucleotide sugar metabolism	9.54×10^{-5}
		map00061	Fatty acid biosynthesis	0.000
		map00511	Other glycan degradation	0.000
		map02024	Quorum sensing	0.001
Green (N=37)	Metabolic and Social Adaptation for Microbial Survival	map02024	Quorum sensing	3.36×10^{-6}
		map00052	Galactose metabolism	0.003
		map01230	Biosynthesis of amino acids	0.010
		map00051	Fructose and mannose metabolism	0.013
		map00500	Starch and sucrose metabolism	0.040
Yellow (N=38)	Carbohydrate-Driven Community Metabolism and Signaling	map00500	Starch and sucrose metabolism	5.69×10^{-6}
		map00052	Galactose metabolism	5.69×10^{-6}
		map00520	Amino sugar and nucleotide sugar metabolism	0.001
		map02024	Quorum sensing	0.001

* Kyoto Encyclopedia of Genes and Genomes (KEGG) Top 5 based on the adjusted P value for each term.

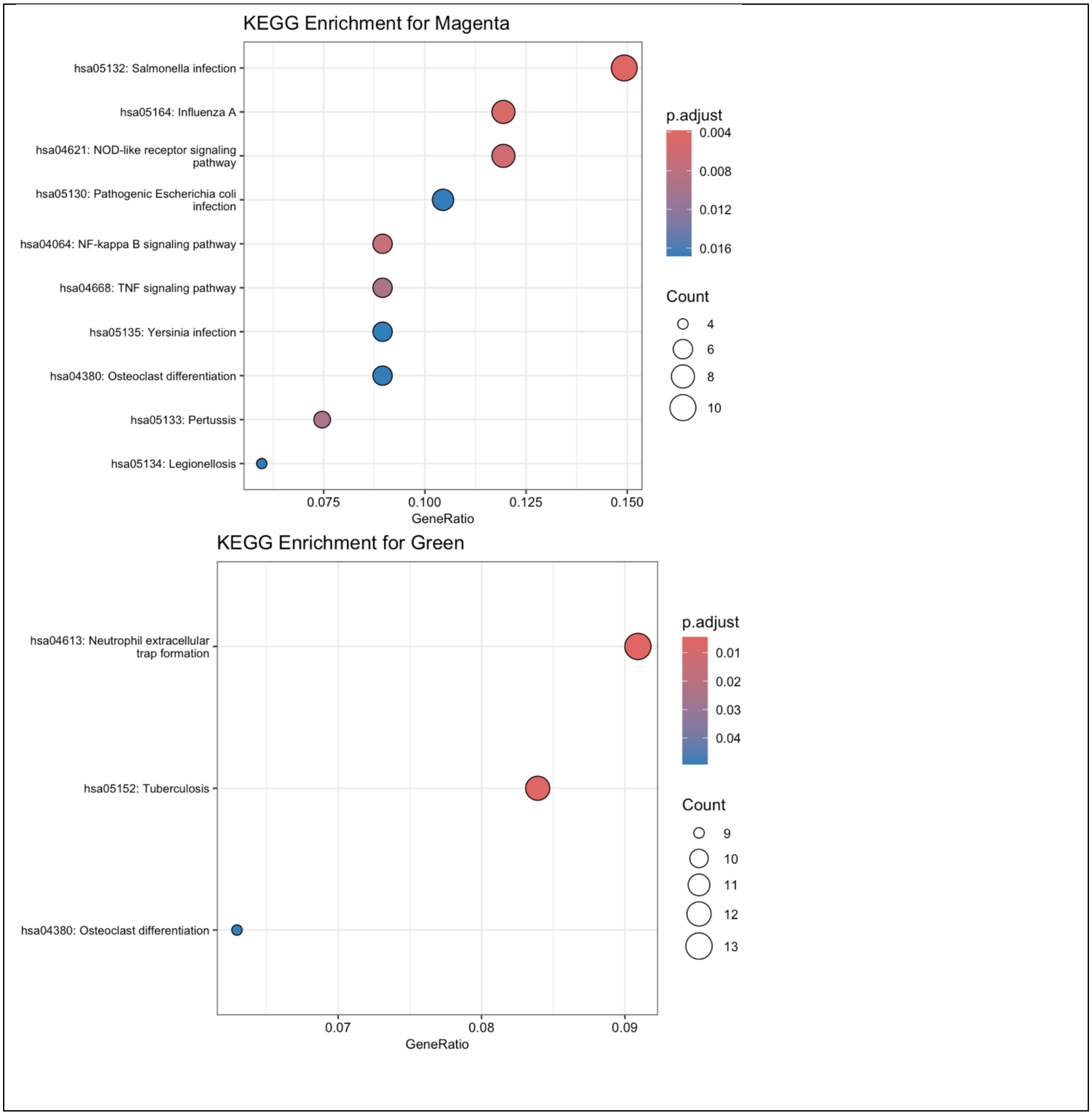
KEGG Enrichment for Magenta
hsa05132: Salmonella infection
hsa05164: Influenza A
hsa04621: NOD-like receptor signaling pathway
hsa05130: Pathogenic Escherichia coli infection
hsa04064: NF-kappa B signaling pathway
hsa04668: TNF signaling pathway
hsa05135: Yersinia infection
hsa04380: Osteoclast differentiation
hsa05133: Pertussis
hsa05134: Legionellosis
0.075
0.100
0.125
0.150
GeneRatio
p.adjust
0.004
0.008
0.012
0.016
Count
4
6
8
10
KEGG Enrichment for Green
hsa04613: Neutrophil extracellular trap formation
hsa05152: Tuberculosis
hsa04380: Osteoclast differentiation
0.07
0.08
0.09
GeneRatio
p.adjust
0.01
0.02
0.03
0.04
Count
9
10
11
12
13

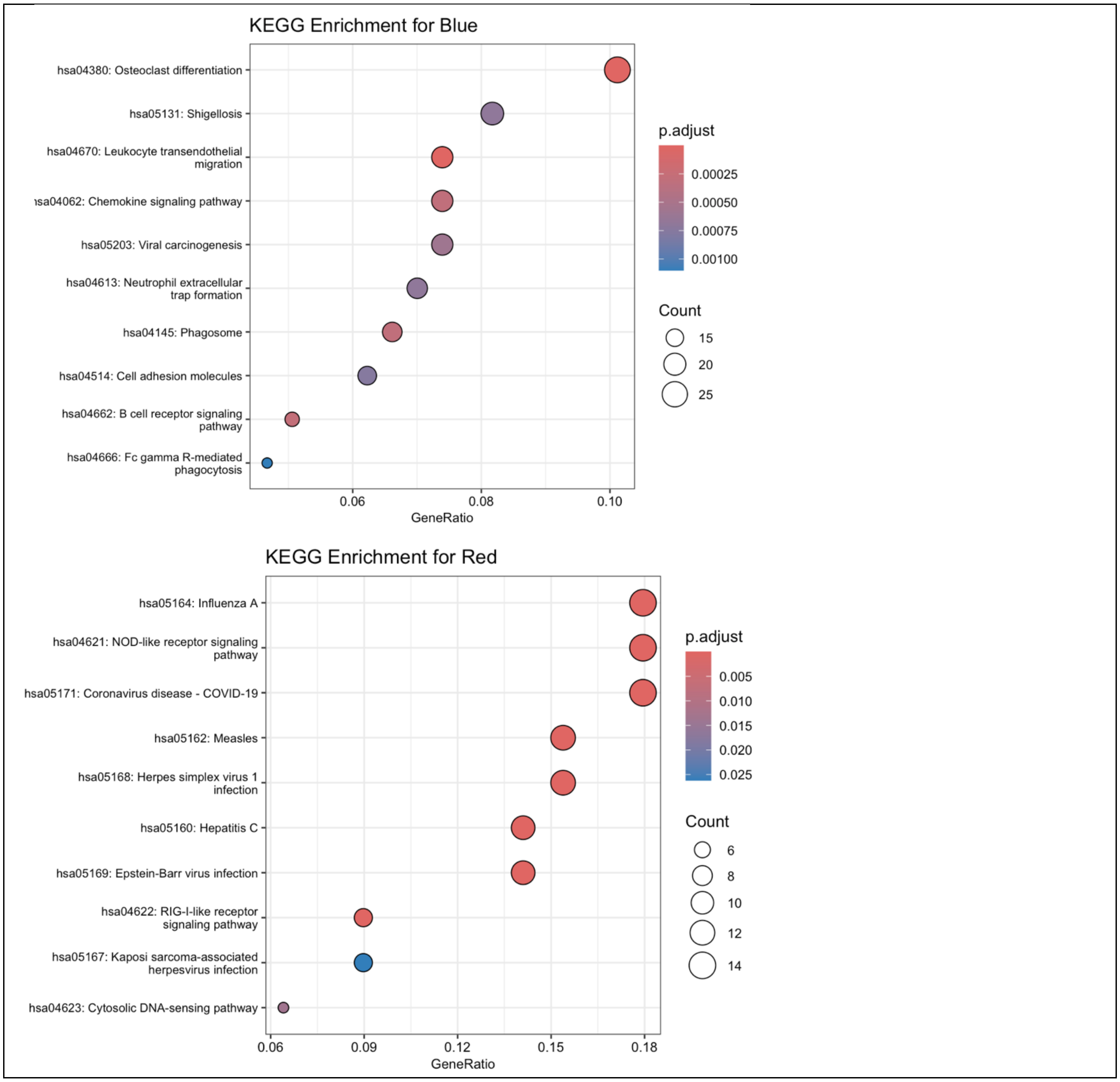
KEGG Enrichment for Blue
hsa04380: Osteoclast differentiation
hsa05131: Shigellosis
hsa04670: Leukocyte transendothelial migration
hsa04062: Chemokine signaling pathway
hsa05203: Viral carcinogenesis
hsa04613: Neutrophil extracellular trap formation
hsa04145: Phagosome
hsa04514: Cell adhesion molecules
hsa04662: B cell receptor signaling pathway
hsa04666: Fc gamma R-mediated phagocytosis
0.06
0.08
0.10
GeneRatio
p.adjust
0.00025
0.00050
0.00075
0.00100
Count
15
20
25
KEGG Enrichment for Red
hsa05164: Influenza A
hsa04621: NOD-like receptor signaling pathway
hsa05171: Coronavirus disease - COVID-19
hsa05162: Measles
hsa05168: Herpes simplex virus 1 infection
hsa05160: Hepatitis C
hsa05169: Epstein-Barr virus infection
hsa04622: RIG-I-like receptor signaling pathway
hsa05167: Kaposi sarcoma-associated herpesvirus infection
hsa04623: Cytosolic DNA-sensing pathway
0.06
0.09
0.12
0.15
0.18
GeneRatio
p.adjust
0.005
0.010
0.015
0.020
0.025
Count
6
8
10
12
14

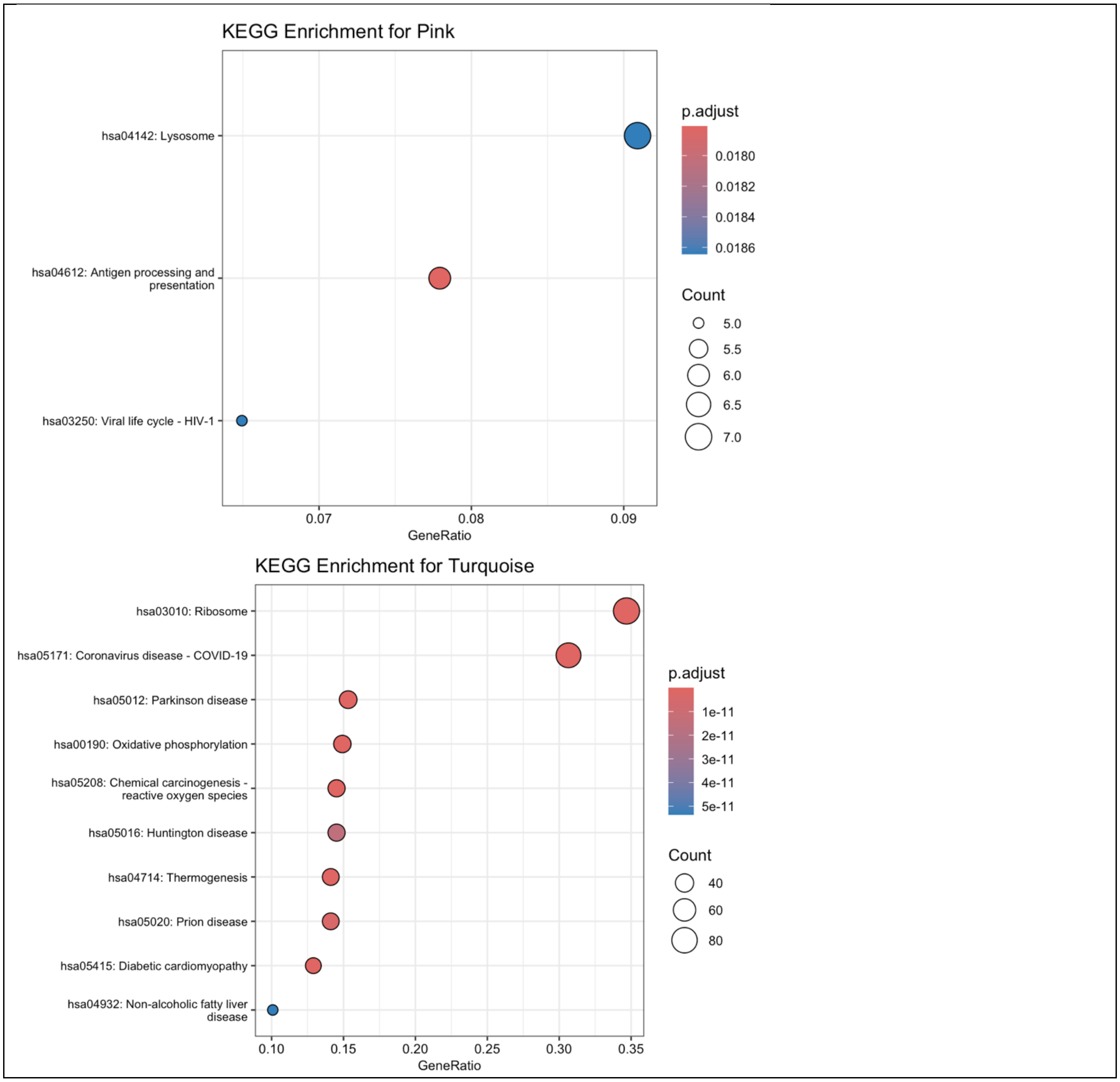
KEGG Enrichment for Pink
hsa04142: Lysosome
hsa04612: Antigen processing and presentation
hsa03250: Viral life cycle - HIV-1
0.07
0.08
0.09
GeneRatio
p.adjust
0.0180
0.0182
0.0184
0.0186
Count
5.0
5.5
6.0
6.5
7.0
KEGG Enrichment for Turquoise
hsa03010: Ribosome
hsa05171: Coronavirus disease - COVID-19
hsa05012: Parkinson disease
hsa00190: Oxidative phosphorylation
hsa05208: Chemical carcinogenesis - reactive oxygen species
hsa05016: Huntington disease
hsa04714: Thermogenesis
hsa05020: Prion disease
hsa05415: Diabetic cardiomyopathy
hsa04932: Non-alcoholic fatty liver disease
0.10
0.15
0.20
0.25
0.30
0.35
GeneRatio
p.adjust
1e-11
2e-11
3e-11
4e-11
5e-11
Count
40
60
80

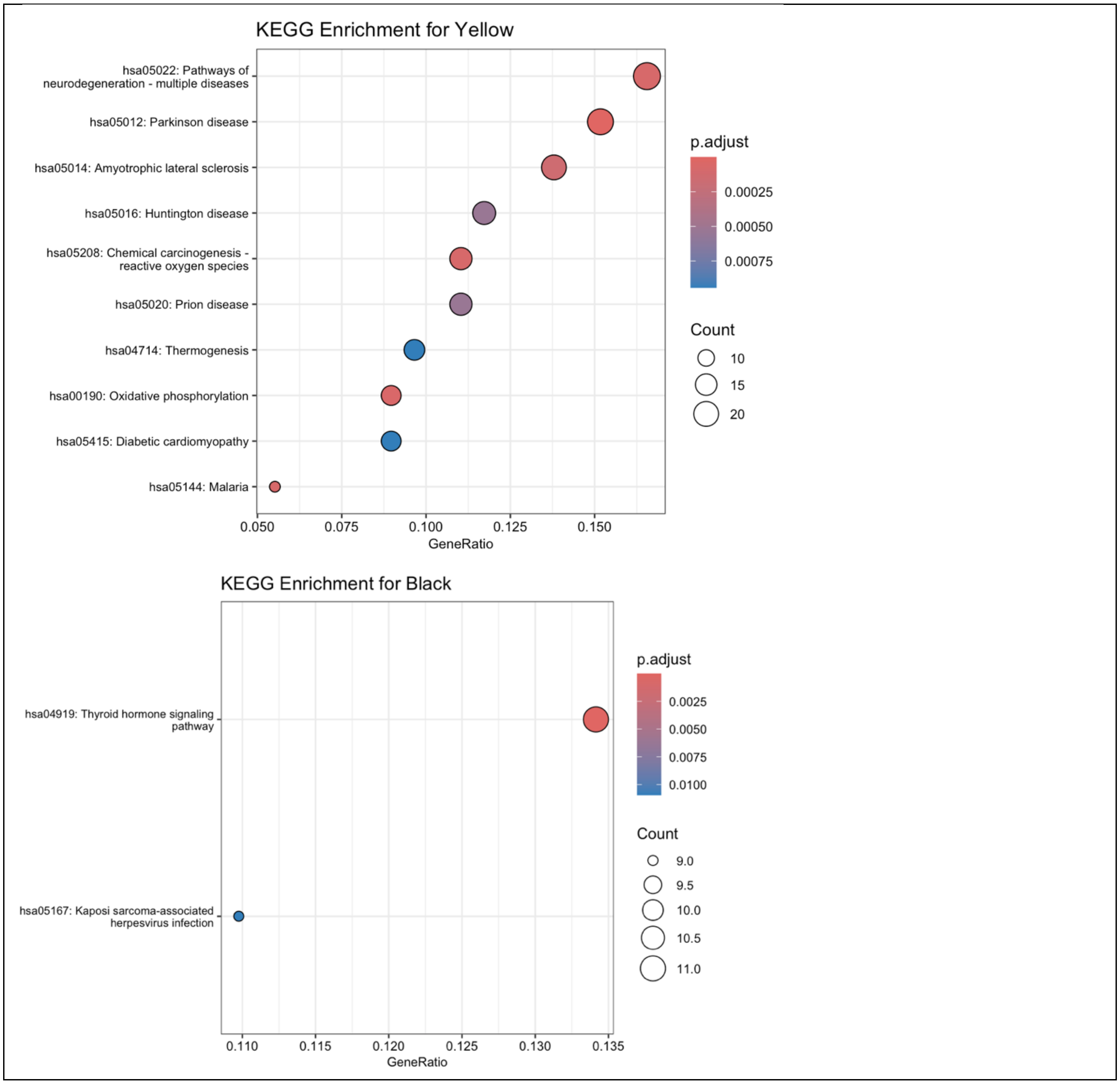
KEGG Enrichment for Yellow
hsa05022: Pathways of neurodegeneration - multiple diseases
hsa05012: Parkinson disease
hsa05014: Amyotrophic lateral sclerosis
hsa05016: Huntington disease
hsa05208: Chemical carcinogenesis - reactive oxygen species
hsa05020: Prion disease
hsa04714: Thermogenesis
hsa00190: Oxidative phosphorylation
hsa05415: Diabetic cardiomyopathy
hsa05144: Malaria
0.050
0.075
0.100
0.125
0.150
GeneRatio
p.adjust
0.00025
0.00050
0.00075
Count
10
15
20
KEGG Enrichment for Black
hsa04919: Thyroid hormone signaling pathway
hsa05167: Kaposi sarcoma-associated herpesvirus infection
0.110
0.115
0.120
0.125
0.130
0.135
GeneRatio
p.adjust
0.0025
0.0050
0.0075
0.0100
Count
9.0
9.5
10.0
10.5
11.0

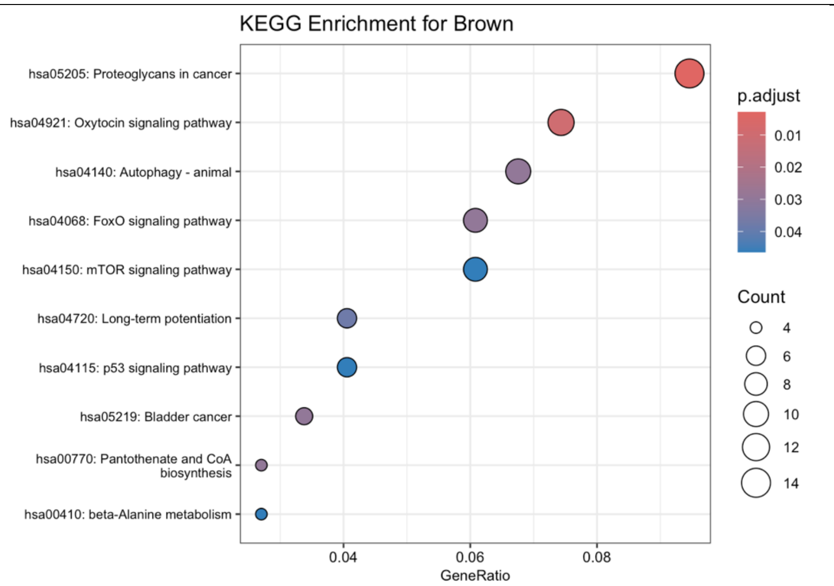

Figure S1. The Kyoto Encyclopedia of Genes and Genomes (KEGG) Pathway Enrichment Analysis of Transcriptomic Modules. The size of each bubble corresponds to the number of genes associated with each KEGG pathway, with larger bubbles indicating a higher gene count. The color gradient represents the significance of enrichment based on adjusted p-values (FDR < 0.05).

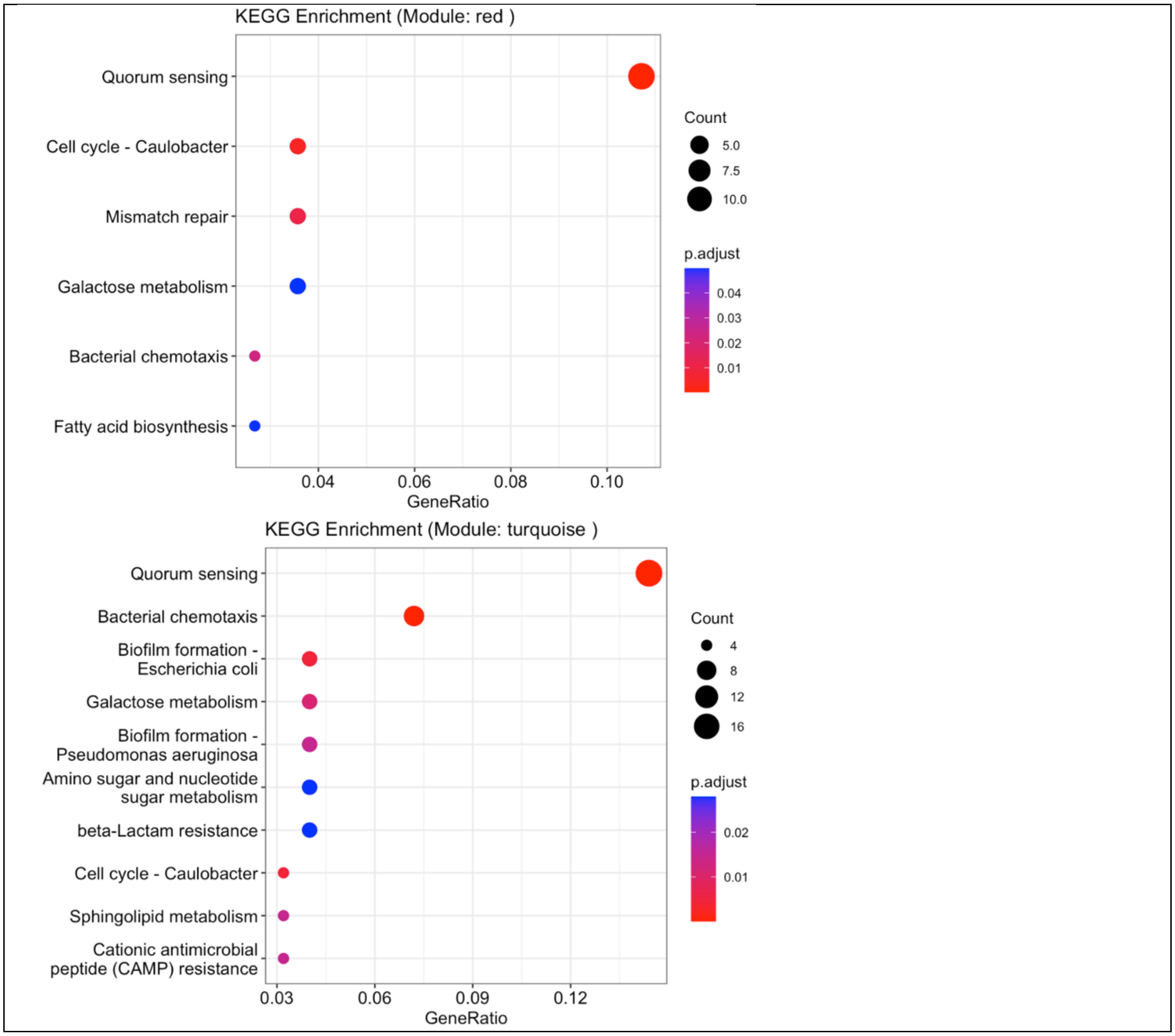
KEGG Enrichment (Module: red)
Quorum sensing
Cell cycle - Caulobacter
Mismatch repair
Galactose metabolism
Bacterial chemotaxis
Fatty acid biosynthesis
0.04
0.06
0.08
0.10
GeneRatio
Count
5.0
7.5
10.0
p.adjust
0.04
0.03
0.02
0.01
KEGG Enrichment (Module: turquoise)
Quorum sensing
Bacterial chemotaxis
Biofilm formation - Escherichia coli
Galactose metabolism
Biofilm formation - Pseudomonas aeruginosa
Amino sugar and nucleotide sugar metabolism
beta-Lactam resistance
Cell cycle - Caulobacter
Sphingolipid metabolism
Cationic antimicrobial peptide (CAMP) resistance
0.03
0.06
0.09
0.12
GeneRatio
Count
4
8
12
16
p.adjust
0.02
0.01

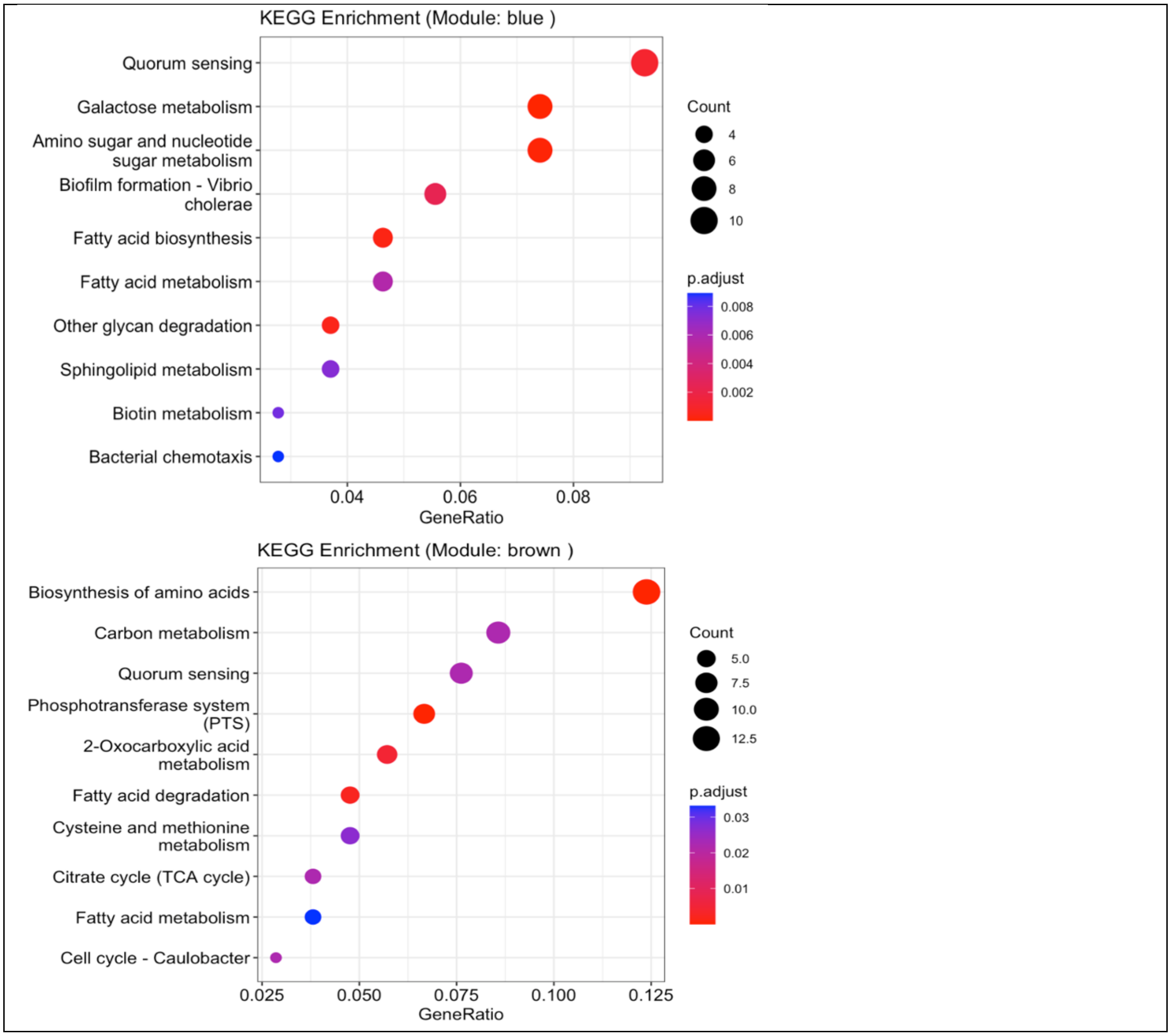

KEGG Enrichment (Module: blue)
Quorum sensing
Galactose metabolism
Amino sugar and nucleotide sugar metabolism
Biofilm formation - Vibrio cholerae
Fatty acid biosynthesis
Fatty acid metabolism
Other glycan degradation
Sphingolipid metabolism
Biotin metabolism
Bacterial chemotaxis
0.04
0.06
0.08
GeneRatio
Count
4
6
8
10
p.adjust
0.008
0.006
0.004
0.002
KEGG Enrichment (Module: brown)
Biosynthesis of amino acids
Carbon metabolism
Quorum sensing
Phosphotransferase system (PTS)
2-Oxocarboxylic acid metabolism
Fatty acid degradation
Cysteine and methionine metabolism
Citrate cycle (TCA cycle)
Fatty acid metabolism
Cell cycle - Caulobacter
0.025
0.050
0.075
0.100
0.125
GeneRatio
Count
5.0
7.5
10.0
12.5
p.adjust
0.03
0.02
0.01

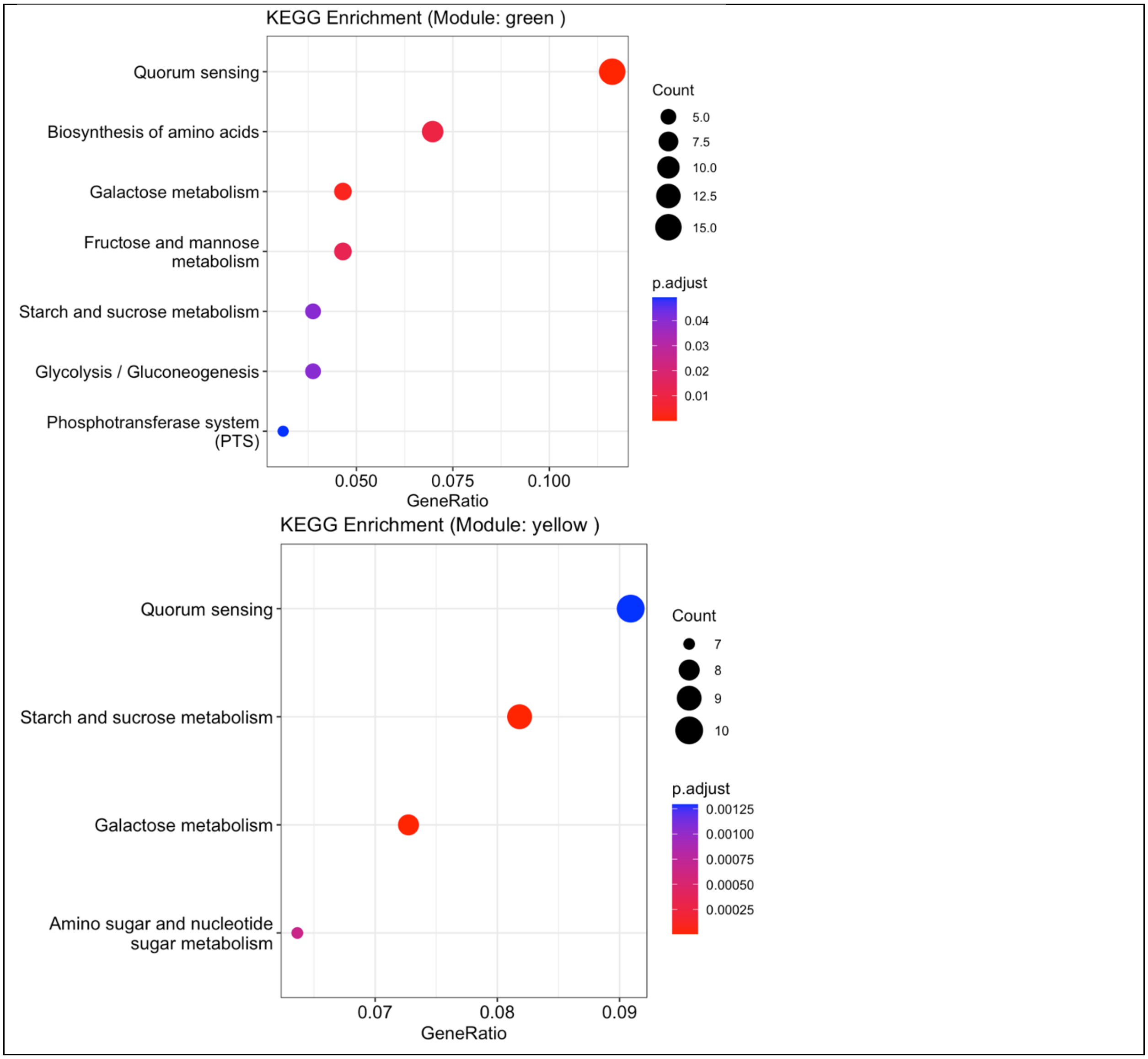
KEGG Enrichment (Module: green)
Quorum sensing
Biosynthesis of amino acids
Galactose metabolism
Fructose and mannose metabolism
Starch and sucrose metabolism
Glycolysis / Gluconeogenesis
Phosphotransferase system (PTS)
0.050
0.075
0.100
GeneRatio
Count
5.0
7.5
10.0
12.5
15.0
p.adjust
0.04
0.03
0.02
0.01
KEGG Enrichment (Module: yellow)
Quorum sensing
Starch and sucrose metabolism
Galactose metabolism
Amino sugar and nucleotide sugar metabolism
0.07
0.08
0.09
GeneRatio
Count
7
8
9
10
p.adjust
0.00125
0.00100
0.00075
0.00050
0.00025

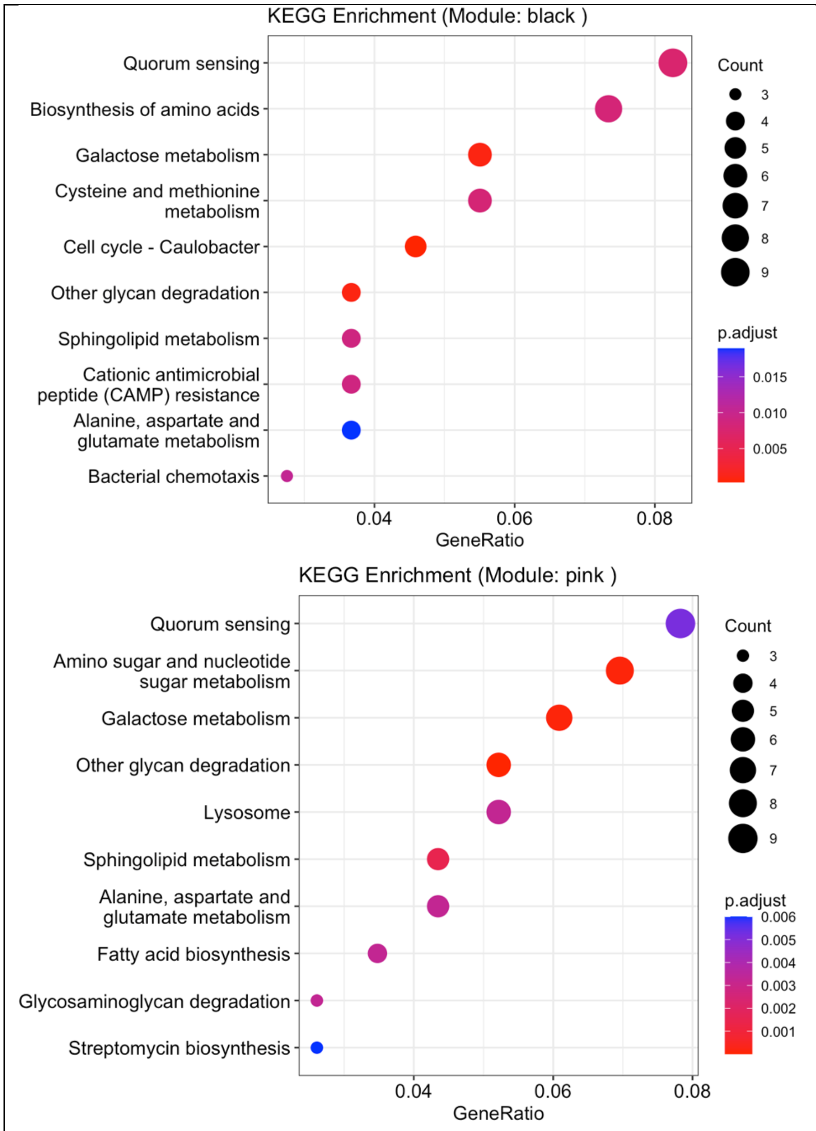

Figure S2. The Kyoto Encyclopedia of Genes and Genomes (KEGG) Pathway Enrichment Analysis of Microbiome Modules. The size of each bubble corresponds to the abundance associated with each KEGG pathway, with larger bubbles indicating a higher abundance. The color gradient represents the significance of enrichment based on adjusted p-values (FDR < 0.05).
